# Octopus: A Design Methodology for Motion Capture Wearables

**DOI:** 10.3390/s17081875

**Published:** 2017-08-15

**Authors:** Javier Marin, Teresa Blanco, Jose J. Marin

**Affiliations:** 1 IDERGO (Research and Development in Ergonomics) Research Group, I3A (Aragon Institute of Engineering Research), University of Zaragoza, C/Mariano Esquillor s/n, 50018 Zaragoza, Spain; 647473@unizar.es; 2 HOWLab (Human Openware Research Lab) Research Group, I3A (Aragon Institute of Engineering Research), University of Zaragoza, C/Mariano Esquillor s/n, 50018 Zaragoza, Spain; tblanco@unizar.es; 3 Department of Design and Manufacturing Engineering, University of Zaragoza, C/María de Luna, 3, 50018 Zaragoza, Spain

**Keywords:** design methodology, design requirements, wearables, MoCap, body positioning, body attachment, IMU, rigid bodies

## Abstract

Human motion capture (MoCap) is widely recognised for its usefulness and application in different fields, such as health, sports, and leisure; therefore, its inclusion in current wearables (MoCap-wearables) is increasing, and it may be very useful in a context of intelligent objects interconnected with each other and to the cloud in the Internet of Things (IoT). However, capturing human movement adequately requires addressing difficult-to-satisfy requirements, which means that the applications that are possible with this technology are held back by a series of accessibility barriers, some technological and some regarding usability. To overcome these barriers and generate products with greater wearability that are more efficient and accessible, factors are compiled through a review of publications and market research. The result of this analysis is a design methodology called Octopus, which ranks these factors and schematises them. Octopus provides a tool that can help define design requirements for multidisciplinary teams, generating a common framework and offering a new method of communication between them.

## 1. Introduction

Movement is healthy, but whether from injury, illness, or ageing, we are all exposed to losing motor skills at some stage in our lives. In that case, we need to carry out a process of re-education, training, or rehabilitation that teaches us to move or exercise in a certain way. In this sense, motion capture (MoCap) is an opportunity that provides information, both directly (range of mobility of a joint) and indirectly (habits, physical inactivity, etc.) [1,2].

The exponential rate of technology development, and its extensive access through platforms, such as Arduino or Raspberry-Pi, has fostered a world of interconnected objects and connection to the cloud and the Internet of Things (IoT) [3]. In this context, existing objects take on new features, and new ones are inspired by the technology itself. In this way, the elements that we place in the body undergo this evolution, giving rise to a new generation of products, the wearables, whose appearance can be considered at the conceptual level as well as the user acceptance level, as one of the small revolutions within the IoT.

Based on the criteria proposed by Knight et al. [4], wearables can be defined as devices that allow user interaction and user data collection, while remaining attached to the body, regardless of the body’s activity and without muscular effort required to hold them. Additionally, wearability describes the interaction between the human body and the wearable device, and dynamic wearability includes the movement of the human body in the design [5].

For this text, the devices intended to capture human movement that meet the above-mentioned characteristics of wearability and dynamic wearability concepts will be referred to as motion capture wearables (MoCap-wearables). Specifically, we refer to MoCap systems based on the rigid bodies, concept defined by Skogstad et al. [6] as objects that will not deform and will simulate or monitor a body segment.

**Figure 1:**
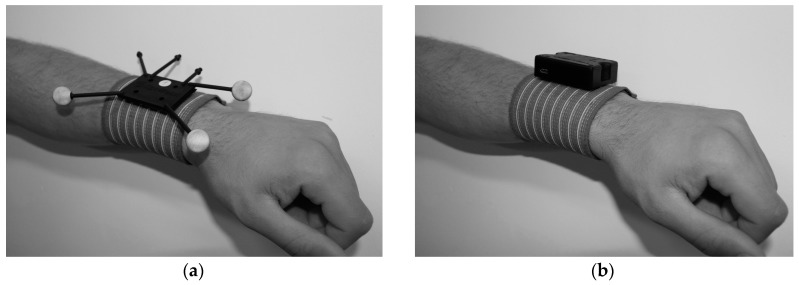
Motion capture (MoCap) rigid bodies: (**a**) optical rigid body [14]; (**b**) IMU rigid body [15].

As shown in Figure 1, MoCap systems that are based on rigid bodies correspond to clusters of reflective spherical markers (Figure 1a) that can be univocally identified by infrared light emitting cameras [6,7], providing three rotations and three positions (one for each space axis). They can also be electronic devices—inertial measurement units (IMUs, Figure 1b)—that provide rotations (rotation matrix, euler angles, quaternions, etc.) through signal processing of the output data of different built-in sensors accelerometers, gyroscopes, and magnetometers) [8,9,10]. Furthermore by processing from the IMU acceleration data, more information can be extracted, such as speed and position [11,12] or even the moment of reaction on the ground [13]. For MoCap, the concept of a rigid body, in which we focus, matches for both cases, to associate a rigid body to a body segment.

Currently, MoCap applications are mainly restricted to research, medical rehabilitation, sports training, augmented reality systems, and 3D animation [7,16]. Unfortunately, the large numbers of applications that are possible with this technology are held back by many barriers related to various factors. Overcoming such barriers, we could improve existing applications through more usable, democratised, and higher quality technology. Alternatively, we can generate innovative methods of using MoCap through disruptive innovations based on its application in new fields.

There are barriers associated with errors of the technology itself (magnetic field disturbances, gyroscope drift, etc.) that have had a clear effect on the effectiveness of MoCap systems and therefore on the trust of the technology associated with them [17]. However, due to advances in technology, we observe that the errors directly linked to it are progressively diminishing, either through new or better signal processing or hardware upgrades [10,18,19,20,21].

Nevertheless, there are other barriers that are currently unavoidable, such as difficulty in measuring the skin, muscles, and soft tissue movements around the bones. This is one of the most problematic error sources in MoCap systems that use optical or IMU surface markers [20,22]. According to Capozzo et al. [23], isolated markers located directly on the skin at specific anatomical points (landmarks) may undergo relative displacements to the underlying bone in the range of 10–30 mm, which causes an error accumulation in the segment angle. Thus, it should be assumed that MoCap systems with surface markers do not represent bone movement [24].

On this basis, one of the main barrier in terms of error to overcome in MoCap applications is reproducibility [22,25]. Therefore, the accuracy of the body segment angle measurement does not matter, as the same results are obtained using a certain MoCap system with the same subject on different days with different operators in independent laboratories.

The reproducibility factor in a MoCap system is conditioned by the attachment of the markers or sensors to the body [20]. If the union is not constant, stable, and rigid, the measurement quality may worsen through the existence of relative movements between the devices and body [25,26,27]. Various authors have referred to errors derived for this reason in their research [6,8,11,28]. Although some authors consider the position of the IMUs in the body [29,30], positioning continues to be performed only by a visual check, by trying to align sensor axes with the segment [27,28,31]. Despite being decisive, union elements between the electronic device (IMU) and the body are usually one of the last issues considered in the design, when this should be a matter considered during product design.

We are therefore facing a design challenge, a problem that has been asserted by multiple authors and that transcends reproducibility and measurement accuracy. In this way, to offer products that expand the current sectorial limits (health, sports, 3D animation, etc.), factors such as body attachment, usability, device comfort [9], or accessible costs among others, should be considered, as these are the keys to an optimum MoCap-wearable design.

In view of these problems, this article collects design principles related to the physical aspects of MoCap-wearable systems. The challenge is to identify all those factors that affect the creation process to achieve a MoCap system design that includes both the most purely technical requirements and those that are more focused on the users and the environments. Collected factors are ranked and schematised in a design methodology for requirement definitions called Octopus. The aim of this tool is to facilitate and guide designers and other professionals involved in the development by generating knowledge that allows precisely considering, without omission, all factors from the initial stages of development. The tool is expected to improve existing applications or support the creation of new ones.

## 2. Materials and Methods

In the wake of the design needs detected, considering the high complexity of MoCap-wearable systems, this section presents a series of factors that are indispensable for study in the design process. These factors have been grouped by theme into five sub-sections: context (Section 2.1), technology (Section 2.2), body attachment (Section 2.3), physical properties (Section 2.4), and user interaction (Section 2.5).

A two-line research methodology has been carried out to identify the design factors. On one hand, a literature review has been conducted regarding wearables and MoCap, these articles are cited to describe the factors, and, on the other hand, an extensive study of the products currently available in the market has been done. Regarding this last point, Table 1 shows a selection of those studied products that will facilitate illustrating the factors proposed in this article since they have been considered representative of the different fields of application and/or provide innovative or unique solutions. Both full body MoCap systems and wearables products have interesting and prominent characteristics that have served as reference to find some of the following factors. Although not all selected wearable products are designed for MoCap purposes, we have used them as inspirational products, extrapolating the characteristics to the MoCap field.

For terminology in this document, *devices* will be understood as MoCap elements to be placed on the body and data processing points (DPPs) will be understood as elements to which they can be connected, such as computers, smartphones, tablets, consoles, etc. DPPs will carry out certain actions with the information collected from the devices. In this way, a set of devices, DPP, and the operation of use constitutes a system, which may refer to commercial systems (wearables or MoCap) or those that are intended to be designed (MoCap-wearables).

### 2.1. Contextual Factors

Contextual awareness addresses the study of scenarios in which the product is used [60] and allows developers to focus design decisions on the user’s world, achieving greater acceptance and system usability. The contextual characteristics will translate into requirements and design opportunities, which allow cross-functional decisions that affect other factors. This section includes factors related to use, the user, and the environment of the product to be designed.

Environmental studies can provide information about available resources, protocols of use, level of hygiene required, etc., while user studies may allow considering both explicitly as latent needs related to psychological, physical, behavioural, or formative aspects.

Generally, and specifically in the case of MoCap-wearables systems, the environment defines the user. Table 2 lists the fields of existing applications—in line with Ahmad et al. [16] and Mayagoitia et al. [7]—and the users involved in each of them. Differentiation is made between two user profiles that can be identified in a MoCap-wearable system: the professional user, which is the person interested in obtaining the data and usually is the product purchaser or prescriber, and the actor user, which is the person captured by the system who may be interested in obtaining information. We assume that the concept of user profile may include one or more individuals as appropriate.

As seen in Table 2, in the medical and sports fields, the professional user may be a doctor, physiotherapist, or coach and the actor user a patient or athlete. In this area, patients are usually collaborators, except in the forensic environment, where expectations of a possible financial compensation for the damage or injury caused by an accident or other cause may call into question such collaboration. In 3D animation and simulation, user types depend on each application; for example, in video games, it is singular that the profiles of the professional and actor coincide in the same person: the player. Finally, in the research sector, professional users are researchers or developers who are interested in obtaining information or implementing new applications.

**Figure 2:**
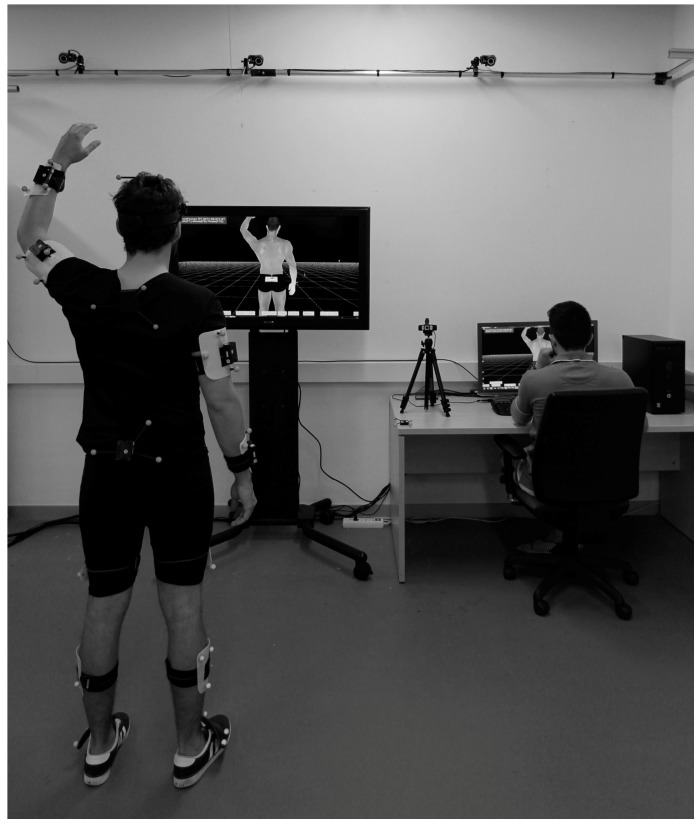
MoCap service, biomechanics laboratory. System with simultaneous optical and IMU technology (optical full body MoCap and IMU upper body MoCap).

It should be noted that, according to the mode of application or use of the system, the resulting niche can lead not only to a new commercial product development but also to a service or a product-service operation. Some manufacturers are already marketing MoCap systems as a service. This is the case of MySwing [34], where the customer has a personalised learning and analysis program of his or her golf practice. Likewise, a MoCap professional studio for the film industry or a gait analysis laboratory is intrinsically a service [41,47]. Figure 2 illustrates a real MoCap service with both described users involved.

It is important to note that if a MoCap-wearable design is associated with a service, the system profitability and its purchase interest may depend on the developer or customer ability to perform an adequate servitisation of the product. In addition, the training, motivation, and resources available to the professional will be keys for the service to be adequate. In addition, the privacy and the required confidentiality level in each of the usage scenarios must be considered [60,61].

Therefore, assuming the business model as a service, defining the operational use from the initial development stages will allow applying specific design techniques to predict failures, extracting critical points and accompanying the physical design with the considerations related to the intangible part of the product.

### 2.2. User Interaction Factors

Achieving an appropriate interaction between users and the product is relevant to minimise the learning phase and avoid errors during use. In the literature review, it has been detected that, as a general rule, some adjectives that define a good user interaction with wearable devices are simplicity, subtlety, transparency, and intuitiveness [5,60].

The product-service system can interact with both the professional user and the actor user. Therefore, the information to be transmitted and its representation must be adequate to each profile mental model and to the context at the time of receiving the information [62]. Therefore, the user characterisation will greatly influence the interaction design, which will be a cross-functional theme for all product factors.

**Figure 3:**
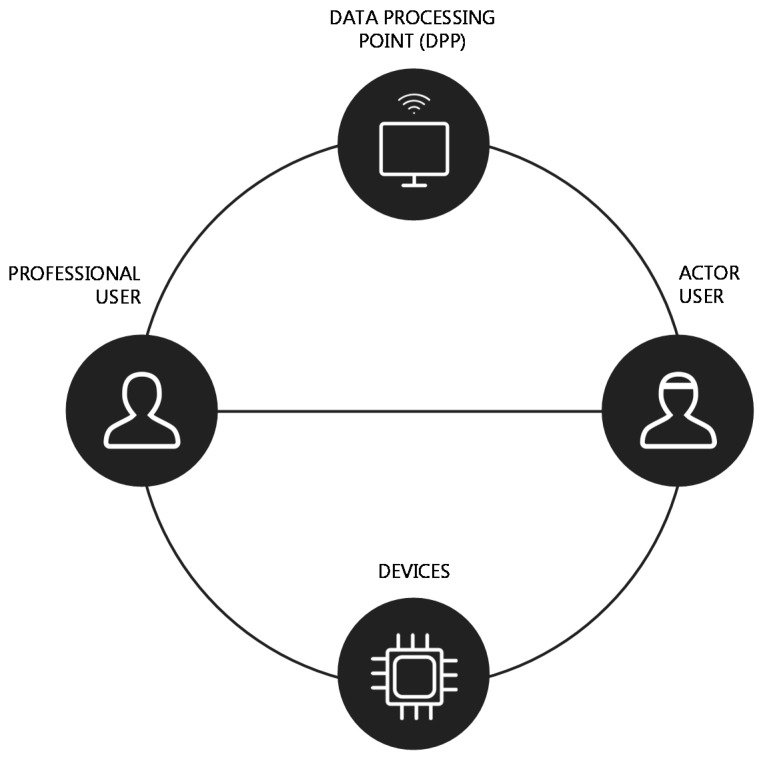
Communication interfaces scheme in a MoCap service. (Icons designed by Freepik and Alfredo Hernandez, from www.flaticon.com).

To classify the communication interfaces between users and technology, the two parts of the MoCap-wearable system can be used: devices and DPP. In Figure 3, the communication interfaces are illustrated: users with device and users with DPP. Note that there is also interaction between both types of users, but the definition of such interaction depends on the service design already discussed in Section 2.1.

#### 2.2.1. User Interface with MoCap-Wearable Device

MoCap-wearable devices require a user interface that should allow, facilitate, and optimise product usage, helping its cognitive interpretation. In the reviewed devices (Table 1), there are physical interaction components (power button, load connector, and light-emitting diodes (LEDs) to show the battery level and/or wireless connection status), and graphical components (X, Y, and Z axes on the surface to the device orientation in the body, and body segment labels). These interaction elements are generally aimed to the professional user; however, device designs should consider and take advantage of communication opportunities with the two types of users involved.

Through the interaction elements or other elements that can be incorporated in a bidirectional interaction process, the devices will be able to receive input stimuli (subject movements, change of configuration, etc.) and respond by feedback or output to the users. Such feedback can serve to communicate with the actor through biofeedback [63] (guide, correct movements, etc.) or to reach agreement with the professional (improper device positioning, wrong movements, low battery, etc.). The feedback types can be classified by human senses involved in communication:

- *Visual feedback*: Interaction with LEDs, images, or text. From the perspective of the user actor, an interesting point of interaction may be the upper face of the wrist, inspired by wearable wristwatch style designs or visible areas of the body, such as legs or arms.
- *Aural feedback*: Sounds, beeps, instructions, etc.
- *Haptic feedback*: Communication through non-visual and non-auditory sensations using vibration, temperature, or electrical impulses, which can be observed in some products, such as the Notch motion sensor [38], Araig jacket [36], or the Tesla suit [54].

#### 2.2.2. User Interface with DPP

The DPP can also interact with both users at both hardware and software levels. From the hardware point of view, the most common design process is to select a smart device, such as a computer, smartphone, tablet, game console, etc. This can be recognised in most analysed products. Generally, in MoCap systems, a computer is employed, and in wearables, smartphone use is more frequent. The information input type (touch, voice, etc.) and the output characteristics (visual, sound, etc.) will depend on the selected DPP features to which the designer must adapt.

With this, the problem can be reduced to a software design issue and, from the standpoint of interaction, can be reduced to the screen interface or graphic user interface (GUI) design. Additionally, the software may be able to be supported in the cloud or even only operate on it; thus, the Internet communication will serve to store and download information or communicate with other systems.

### 2.3. Technological Factors

Technology, its possible configurations, and the main characteristics of the electronic components influence the device external parts that are in contact with the user [5]. Consequently, the technological requirement study includes the selection between optical or inertial systems, the electronic component structure that incorporates each capture device, and the possible configurations of interconnection between them.

#### 2.3.1. Choice between Optical and Inertial MoCap Technology

The choice between inertial or optical MoCap technology depends on the application requirements, which should be contrasted with the characteristics of each technology features. In this paper, we focus on these products as they are the most common; however, it should be noted that these are not the only technologies used in MoCap systems [64].

Optical systems have a consolidated development and provide proven accuracy. However, they require a controlled space and environment, especially about lighting (preferably artificial), and a variable number of cameras that will depend on the area of the body to be monitored and the number of simultaneous actors to capture. Normally, the technology is based on vision cameras that emit infrared light, which are capable of recording and processing the movement of reflective spherical markers placed at landmarks [6,7]. Its accuracy is high—on the order of 1% on the measurement taken [65] or 1 mm [22]. It is necessary to emphasise the problems derived from occlusions of the reflective markers by other body parts, other actors, or objects or devices that the actor handles or in the scene itself that may require a high number of cameras or important post-process work to recreate the actor’s movement.

Moreover, inertial systems are generally more economical and require less infrastructure [7]. They can be used either in real time by a DPP signal-receiving device or autonomously with an internal memory storing the information. In contrast, integration errors and the presence of magnetic fields may reduce accuracy [8,10]. For MoCap commercial systems (Table 1), the IMU accuracy offered is in the following intervals: 0.2°–1° (roll/pitch), 0.4°–2° (yaw), and dynamic root mean square (RMS) 1°–2° RMS (roll/pitch/yaw).

#### 2.3.2. Electronic Components (Building Blocks)

It is considered necessary to overcome the strict separation between design engineer and electronic engineer. Knowledge of electronics by the designer allows creation of more viable projects and, alternatively, generates more design opportunities due to the vision of possible technological options. It is true that, for an external device design, it is not necessary to know all the components in depth, but in line with [66], it is interesting to promote designers with minimal technological literacy that are aware and can collaborate in defining the main building blocks. This definition of blocks depends on the usage scenarios (context), especially regarding the times of use, the speed of movements, or the real-time capture needs.

**Figure 4:**
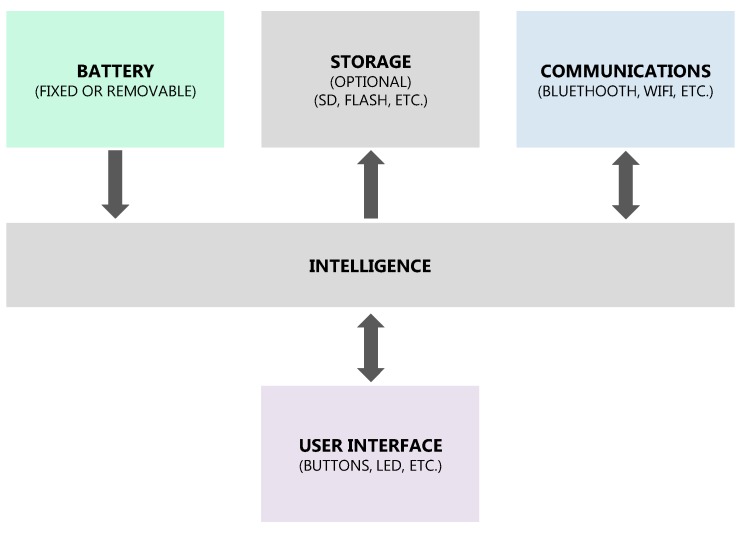
Building blocks example of a MoCap wireless IMU, created with the methodology of Blanco et al. [66].

The building blocks can be considered from the device, the DPP, or even the complete system point of view. In Figure 4, we see an example of the main blocks (building blocks) of a wireless IMU device. Under the scheme, the technological factors to consider for a wireless IMU are related to communication, storage, battery, intelligence, and interface.

In the case of revised IMUs for MoCap-based devices (Table 1), the following factors in relation to electronic components can be highlighted:

- *User interface*: Already mentioned in Section 2.2.
- *Battery*: MoCap wireless sensors typically have built-in non-removable batteries, recharging either in a charging socket or directly connecting to each sensor. The battery life (according to manufacturer’s information) can vary between 3 h and 8 h. The battery selection has a special interrelation with the other factors because it depends on the usage scenarios (life required), on the other components’ consumption, on the DPP characteristics, and on the interconnection between the different components.
- *Storage*: With the option of including internal storage, it is not required to be in range of a wireless network, which increases versatility. In contrast, in this case, the ability to perform real-time processing is limited. Systems that work with internal storage usually have a secure digital (SD) card, such as Perception Neuron or Stt-Systems [33,51].
- *Communication*: The communication features between devices and the DPP depend on the selected wireless communication protocols and consequently on the selected DPP to process the data. However, sometimes, an external dongle communication is connected to the DPP, which relieves its selection requirements. Communication protocols are typically WiFi 2.4–5 GHz for local area networks (LANs), Bluetooth for personal area networks (PANs), or other proprietary protocols.
- *Intelligence*: All these actions are performed by the devices from raw measurement data and users’ actions. In terms of intelligence, the quality of the measurements provided by the IMU, and therefore the restrictions for some applications, depends on the quality in the accelerometers, gyroscopes, and magnetometers and the quality of the signal processing. The IMU measurement ranges vary between ±2 g and ±50 g for accelerometers and between ±150 °/s and ±1000 °/s for gyroscopes [27]; however, the evolution of the technology is improving the quality of these aspects. In signal processing, Kalman filters are widely used [67]. Currently, this field is being improved by different authors. For example, Dejnabadi et al. (2006) and Favre et al. (2009) [19,20] have proposed other magnetic and compensation drift algorithms that have been shown to be effective.

#### 2.3.3. Number of Devices and Interconnection of Them

A key factor in the technology definition is the number of devices be placed on the body areas, each application will require a varying number. The number of sensors influences and depends on the available computational resources and on how much the signal processing can be tailored to the application. In general, for full body MoCap, a total of 15 are required: three per each extremity, another to monitor the head, and two others for the chest. In addition, more sensors can be added in the phalanges or chest. We can see a MoCap application with fewer devices in Macard et al.’s paper [68]. In terms of interconnection and communication between devices, in the studied MoCap systems (Table 1), different possible configurations can be found:

- *Full body suits*: Typically made with Lycra, they contain devices in areas to be monitored. The sensors are wired to a hub placed on the waist or back. The hub communicates wirelessly with the DPP and includes a battery that powers the devices [39,56].
- *Wired independent elements*: These have the same operation and interconnection as the full body suit, but each sensor has an independent fixing support. The cables, sensors, and other devices are in sight [33,46,52].
- *Wireless devices*: Each sensor is placed with an individual fixing support, and each one has its own battery and communicates independently with the DPP [32,34,38,45,51,56].

### 2.4. Body Attachment Factors

Body attachment is one of the key factors in the MoCap-wearable device design, as it is a cross-functional concept. Thus, it directly affects system accuracy, reproducibility, user comfort, and, consequently, product market acceptance [9,25]. Although it could be analysed as a sub-point of the user study (ergonomics), it is considered that it goes further and is considered an independent issue, comprising the good characterisation of a considerable number of requirements.

Body attachment requires studying two sub-factors: positioning and the attachment method. The positioning refers to where the devices are located, that is, in which body segments and in which zones within each segment. The attachment method defines how they are joined, that is, what elements are used to attach the devices to the body segments.

#### 2.4.1. Device Positioning

Yang and Li [27] asserted that the effect of sensor location on measurement quality is not usually analysed; however, the collected data from acceleration and angular velocity are different in one location from another for the same body segment. Consequently, devices should be positioned so that they are oriented towards the body of a subject in the same way regardless of condition, context, or activity. Correct positioning should not depend mainly or exclusively on the professional user; in this sense, the product shape and the rules of use play a key role. Some authors have also detected this fact and have attempted to solve it through different software or hardware improvements [29,30,68,69], which is a complementary approach to the one presented here.

From the point of view of accuracy, to select the position of a body segment regarding where to affix the capture device, the potential effects of that place, and the quality of the movement measurement must be considered. In terms of comfort, devices should generally be non-intrusive and consider the body to be a dynamic structure in motion. Areas with relatively the same size should be selected in adults. Moreover, areas with the least movement, friction, and flexibility when the body is in motion should be selected. The anthropometry of the target users must be considered, and how the sections of each segment change according to age, morphology, and weight should be studied [5,26].

**Figure 5:**
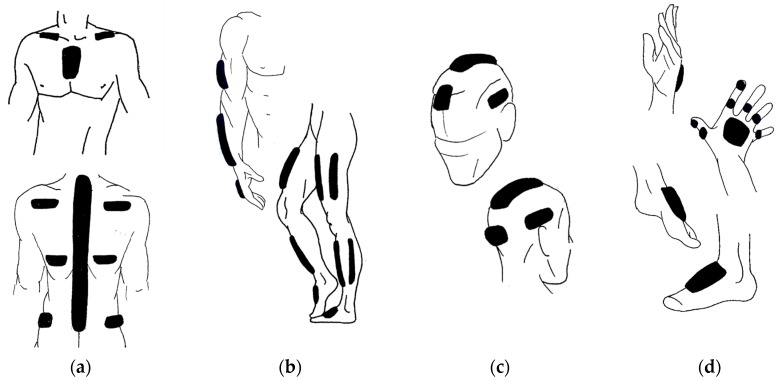
Main zones for positioning devices: (**a**) chest; (**b**) extremities; (**c**) head; (**d**) hands and feet.

Following these rules and analysing the positioning areas of the commercial MoCap devices (Table 1), the most common placement areas for monitoring major segments of the body are shown in Figure 5.

In Figure 5a, areas of chest placement are seen. In this multi-segment zone, we can opt for different positioning alternatives: sternum, spine, hips, ribs, shoulder blades, or clavicles. Most systems place one sensor on the sacrum and another on a higher area of the torso, either the anterior or posterior part. To homogenise the stem analysis, Yu et al. [31] studied the movements of the vertebral column to determine the optimal placement of the sensor placed in the upper part. The results showed that the most representative points to monitor displacement of the medial or lateral trunk were the T7-T8 vertebrae. In these parts, the smallest error occurs (0.5°). However, using more sensors in this area may be beneficial, so some systems place a sensor in the mid-spine zone to improve the approximation of its curvature [32], others also add sensors on the sides (clavicles, shoulder blades, or ribs), which allow monitoring the movements of the shoulders and/or the asymmetrical twists of the body [33,39,45,46,52,56].

Figure 5b shows the placement above the extremities. It should be noted that no consensus has been reached regarding the height at which to place the elements in each segment; nevertheless, there is more agreement about the segment faces used; the outermost zones that do not interfere with the movements are suggested. In the forearm, the upper surface of the wrist is used, just above the joint. In the legs, the frontal or outer zones are used. Sometimes, the same system combines both options, placing elements externally on the thigh and frontally on the shin. A resource that can help positioning the limbs is to take advantage of areas where muscles are inserted into the joint, such as the section between the calf muscle and the knee, which provides a curvature that prevents the device from slipping.

Figure 5c shows areas of head placement, with the most common placement being the front and the back of the head, and sometimes the sides or over the top. It is interesting how the head wearables show other positioning resources, using the natural supports that provide the upper part of the ears and the nasal septum [35,42,53,59].

Additionally, Figure 5d depicts areas where devices are placed on the hands and feet, generally on the top of these body parts. These zones provide a relatively extensive and flat surface without movement impairments. When the fingers are monitored, sensors are placed on each of the proximal phalanges (first segment) and on the distal phalanx (last segment) of the thumb and forefinger.

In relation to the above and from a contextual point of view, a factor related to the positioning that is frequently not considered with the necessary detail is the device interaction with the garments. It is necessary to study what kind of clothes users will wear, the possibility of changing their clothes, and how it will affect their comfort and emotions as well as the intimacy and privacy feeling that may influence the individual acceptance and behaviour. For some applications, it may be necessary to design clothes that are compatible with the devices, either integrating the sensors in them or leaving space for the sensors to be positioned in the appropriate way, all respecting the requirements of hygiene and healthiness.

#### 2.4.2. Device Attachment Methods

Multiple authors have stated the importance of using an appropriate attachment method; this is because union is a key factor in all wearables, but even so more in those that monitor movements [6,8,9,11,25,26,27,28]. In a research environment, the professional can use ad-hoc solutions (adhesive tape, dressings, medical plaster, etc.) that can ensure precise positioning and individual adjustment for each subject. However, in commercial applications, a proposal is required that ensures a constant, stable, rigid, comfortable attachment with minimal preparation requirements [9].

In the MoCap-wearable device body attachment design, it is advisable to try to keep the device as close as possible to the body and bones. In general, it is preferable to secure the device by completely or partially surrounding the body region involved, rather than using single-point fastening systems. Furthermore, the long-term effects of carrying the device should be considered, and its effects on the user from the psychological (comfort) and physical (sweating, tiredness, etc.) points should be analysed. In addition, the diversity of body size should be considered, allowing a certain size and shape customisation [5,60]. Thus, from the reviewed products (Table 1), the following types of body attachment or fixing supports can be distinguished:

- *Fabric fixing supports*: The union with fabric is made from different widths of bands or tape or tight garments. The elastic tape can be closed or open, and the latter will be closed with Velcro, clips, or buckles. The fabric characteristics will influence the union accuracy, perspiration, comfort, and wear resistance. The connection between the fabric and the device can be made with Velcro, pockets, dedicated housing (plastic base), or metal pressure clips that are used by some wearables [38,50]. In addition, it may be beneficial to use wide Velcro areas above the fabric, which allow certain variability at the point of attachment to suit each subject.
- *Disposable adhesive fixing supports*: This support type groups different types of unions: hypoallergenic double-sided adhesive, which is economical although relatively weak, bandage or kinesio-tape, which has high adhesion but with some preparation time required, and disposable electrodes, which are used in some wearables [43,49,58]. The latter can be standard or manufactured specifically for the product, being able to use one or several connection points and allowing measurement of biometric signals. Note that, if we choose disposable adhesive fixing supports, although hygiene is maximum, the cost is higher due to the material expenditure. In addition, body hair and sweating will significantly worsen the adhesion.
- *Semi-rigid fixing supports*: This union type is mainly found in wearable devices that, due to the flexibility of some of the parts, the product stands by itself, wrapping around the body or the garments that the subject wears. This solution is observed in the Thalmic Labs product [55] that has elastic zones to fit the arm, surrounding it as a bracelet, the Jolt Sensor wearable [48] that can be attached to clothing with flexible flaps, or others such as Alex Posture, Google Glass, Melon Headband, or Thync [35,42,53,59] that take advantage of their elasticity to hold onto the head as if they were hair headbands. Although it is a method that does not provide a strong union as others and it may be difficult to apply in all body segments, it must be considered to solve some problems, such as fungible expenditures or hygiene.

It should be noted that the different attachment methods can be combined. In fact, some wearables that use tape to surround body segments combine it with non-slip surfaces [37] or disposable electrodes that adhere the tape to the skin [44].

From these types of bindings, in Table 3, a weighting method for choosing the ideal type in a given application is proposed. It collects the body unions identified in the products and articles reviewed as well as a selection of seven factors that characterise each of them. Assuming that there is no better method of fixing than another, the selection will depend on the weight or value given to each of the seven factors, considering the particularities of each application and context.

The score of each factor varies from 1 to 3, with 1 being the worst and 3 being the best. The ‘Result’ column can be completed by applying Equation (1), whose highest value would correspond to the method of union most recommended for a given application and context:

(1) $$R_{j}=\sum P_{ij}\times W_{i}$$

In Equation (1), *R* is the result obtained by each binding method, *P* is the score assigned to each element of the matrix (1, 2, 3), *W* is the weight assigned to each factor (from 1 to 10), while *j* is the rows and *i* is the columns.

In addition to Table 3 and the attachment methods described, we note that as device fixing support, elements that are already carried by the user in the application environment are a resource to consider. This is the case for some wearables that use everyday items, such as glasses, swimming caps, or jackets [36,42,57]. The use of these elements contains known interaction patterns, which is beneficial for all types of interaction, but it makes special sense when referring to the body attachment factor since it can favour technology learning and confidence. Thus, new union concepts require greater learning time and create a new interaction language; however, this does not mean a worse alternative, as long as the designer considers and plans how to transmit it and teach it to the users.

The most commonly used attachment methods in the reviewed products vary according to the anatomical area involved: on the torso (pelvis and thorax), disposable adhesives, harnesses, or adjustable and elastic tape is used; on extremities, elastic tape or disposable adhesive is used; on the head, hair headbands, elastic tape, helmets, hats, or semi-rigid elements that rest in the anatomical references, such as the ears, nasal septum, front, or nape, are used; on the hands, tape or gloves that surround the thumb and/or fingers to prevent the device slipping are used; and on the feet, disposable adhesive or elements attached to parts of the shoe (cords, tongue, or sole) are used.

### 2.5. Physical Property Factors

The physical properties of the device, such as the shape, dimensions, or weight distribution, affect the user both physically and mentally. Therefore, the needs and restrictions of both user profiles in contact with the product are appreciated even more in this section. These factors have effects on the actor user in the aspects of energy expenditure, biomechanics, posture, movements, and perceived comfort [4]. From the professional user’s point of view, they have effects on comfort, acceptance, use experience, and perceived accuracy.

#### 2.5.1. Shape

The shape is one of the key product elements since it must be intimately linked to its function. The MoCap devices must have forms that facilitate positioning, which helps the professional find the proper fixation area and get and maintain its correct orientation, which are requirements that will vary according to each body part. Furthermore, a smooth and subtle transition must be ensured from the body surface to the device; this can be achieved with a concavity in the inner surface in contact with the body and with convexity in the outer surface to avoid blows and hooks [5]. The studied wearable products (Table 1) mostly follow these guidelines; however, the MoCap IMU devices do not. The latter are generally a rectangular box with slightly rounded corners or other polygonal shapes.

#### 2.5.2. Dimensions

The device dimensions should be adequate to the areas to be monitored and should suit different morphologies, considering the diversity of body sizes [60]. Although the surface they occupy in each body segment is not a critical factor, the thickness is. According to Gemperle et al. [5] there is an intimate space or aura of 0 to 127 mm around the body for the devices, and as a rule, one should try to minimise the thickness as much as possible so that it feels like a part of the body.

#### 2.5.3. Weight

The weight should be distributed so that the maximum load is placed near the body centre of mass [5,60]. In this way, if we have several elements to place, heavier ones should be in the torso, and the lighter ones in the extremities.

#### 2.5.4. Flexibility

It is necessary to consider the flexibility of the MoCap-wearable device components. In this sense, the possibility of integrating flexible electronic-printed circuit boards would be the optimal solution for body adaptation, but this would possibly increase technical complexity and cost. Another option is to use rigid areas coupled with flexible areas. Thus, the flexible areas would be between the solid forms, extending it like wings [5]. This is shown in wearable market products, which integrate rigid areas combined with flexible ones in the same structure [35,43,48,55,59]. The MoCap devices are composed by the IMU zone, are completely rigid, and are near the fixing support area, which is usually more flexible and adaptable. The Shadow MoCap system [46] differs from the others because its sensors are embedded in fabric pads (neoprene type), which makes the entire system flexible.

#### 2.5.5. Material

The material selection depends largely on the context of use, which determines different aspects to consider, such as weather resistance agents, abrasion, impacts, temperature, humidity, required maintenance, washing, hygiene, comfort, and breathability [5,26,60]. The most commonly used material in MoCap device housings is plastic. In wearables, however, we find other materials such as silicone or intelligent fabrics. It is important to note that the use of metallic (ferromagnetic) materials, that alter the magnetic field close to the IMU sensors can disorient its magnetometers, so some manufacturers use a shielded packaging [33].

#### 2.5.6. Comfort

Comfort is not a tangible property by itself, but it depends on the physical aspects, and in MoCap-wearable device design, it is necessary to prioritise this factor [5,60,61] because it increases system usability and acceptance. According to Knight et al. [70], wearable comfort depends on several dimensions or factors: first, from the body attachment, the movement restriction, and the user concern about how the device moves in his or her body. Subsequently, the pain (itching, burning, pricking, heat, etc.), the perceived change (feeling physically different, strange, or uncoordinated), the anxiety that can be caused by the system, and the emotions that the device causes when it is worn; which are issues that link to the next point.

#### 2.5.7. Psychological Aspects

The study of the emotional and psychological aspects that people have towards the products helps deepen understanding of the user and thus should also be present in the design process. According to Spagnolli et al. [61], the users should be characterised according to a series of psychological factors, among which we highlight the attitude towards technology, the perception about the product usefulness, the expected learning effort, the social influence for purchase, and the expected pleasure perception during use.

#### 2.5.8. Aesthetics

In addition, in terms of user comfort, aesthetics can be considered one of the most relevant aspects in the MoCap-wearable device design [5,61]. Aesthetics is a more important issue than it may seem, even more so than in other technological products, because people consciously or unconsciously express themselves with the garments and objects worn, as they define us from the social and relational point of view [71]. In this sense, it may be beneficial to consider the user preferences, interests, and desires, and allow a certain personalisation, as Motti and Caine proposed [55]. Nevertheless, the options at this point are endless depending on the new applications.

## 3. Discussion

MoCap systems have been used for more than two decades, and their usefulness and application in various fields such as health, sports, or leisure are widely recognised; therefore, this is increasing its inclusion in current wearables. However, to adequately capture human motion requires addressing requirements that are difficult to satisfy. Among other issues to consider, devices must maintain their body position independently of the subject movements and not be invasive in order to facilitate natural body movements. With all this and because of its prohibitive costs in the past, MoCap expansion has slowed and unfortunately has been restricted to laboratories or specialised studies.

Today’s society already has broad access to technology (cost reduction, free software development platforms, etc.). Therefore, in the context of interconnected objects (IoT) and constant technological development, it is also necessary to have greater access to the complete process of technology creation (design guidelines, methodologies, etc.). Thus, having tools that help the MoCap-wearables systems development process can introduce an entire range of possibilities and allow extending them to new areas where they are not present or not widespread, solving real problems of users and society.

**Figure 6:**
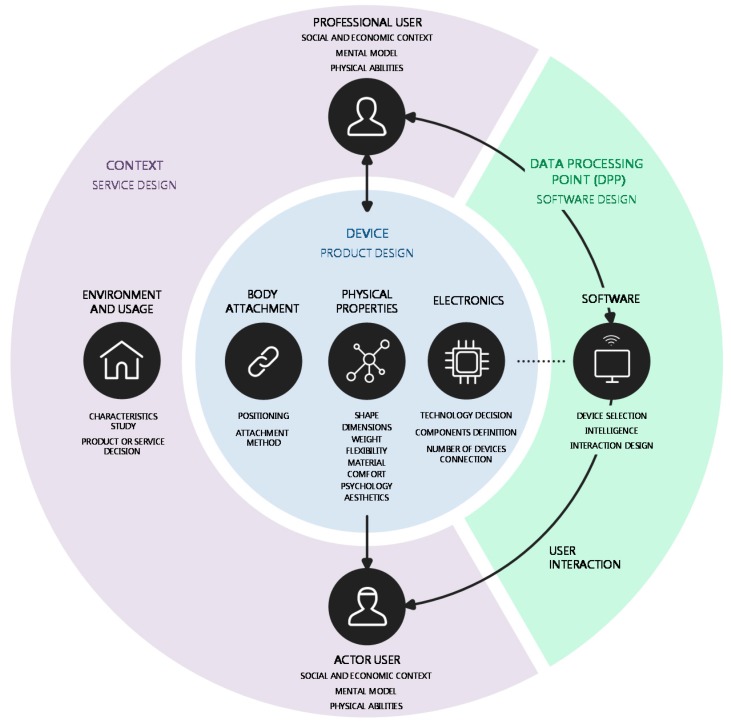
Octopus methodology for MoCap-wearable system designing. (Icons designed by Freepik, recep-kutuk, madebyoliver, gregor-cresnar, EleanorWang, cursor-creative, from www.flaticon.com).

Faced with this problem, a study of the factors that are critical to MoCap-wearable system designs has been conducted. Neglecting any of them may involve creating unprofitable products either because they are not viable or have low effectiveness or low acceptance. In this discussion, a specific design methodology called Octopus is proposed, which aims to manage all these factors and facilitate the requirement definitions of developers, designers, and multidisciplinary design teams. The Octopus is a metaphor for how the devices must be attached to the body, using the eight steps established below. Its scheme is shown in Figure 6.

The Octopus methodology is the result of ad-hoc research and analysis, of the different disciplinary author’s views (mechanical engineering, biomedical, and design), and of the experience in MoCap systems use in the research group, which has conferred a lead user vision that, as Lilien et al. [72] indicated, benefits the design process.

In a general human-centred design methodology, the following iterative studies or stages can be identified: planning, context of use, requirements, design, and evaluation. For each, there are distinctive design methods [73]. The Octopus methodology approaches the first three steps: plan, contextualise, and extract requirements—in this case, the requirements of MoCap-wearable systems.

The scheme in Figure 6 is a representation of the MoCap-wearable product/service and its ecosystem. It is composed of three zones (context, device, and DPP), each of which houses a series of elements directly related to the factors described in the second section (materials and methods). The Octopus methodology proposes to approach the design sequentially, according to the following steps: (1) design goal; (2) context study; (3) service design; (4) user interaction; (5) technology; (6) body attachment; (7) physical properties; and (8) DPP. Although it is linearly represented in principle, it will usually be necessary to perform successive iterations and made a sequence accommodation to each situation.

### 3.1. Step 1: Design Goal

The initial methodology hypothesis starts from the design goal definition. Obviously, the design process evolution—even more in iterative orientations—can slightly or substantially modify this initial objective, especially when one of the main aspirations of the present methodology is linked to the latent need to search for new applications, a necessity for MoCap and wearable evolution. As mentioned, the methodology is flexible enough to adapt to the project imponderables, in line with the iterative design methods, returning to the needed point and taking advantage of the experience gained. In this sense, establishing an abstract character of the design goal will depend on the project philosophy. That is, the challenge can be placed in a concrete improvement (e.g., creating a MoCap system for older people for rehabilitation in their homes) or placing it in more intangible horizons (e.g., look for application fields using new materials). The goal level of abstraction will also influence the starting point, the number of iterations required, and the linear character of the process.

### 3.2. Step 2: Context Study

Once the objective has been established, the next stage is context characterisation, definition and modelling of user types, and use of the product (use, user, and environment analysis). Figure 6 includes the two most common user profiles in MoCap, the professional user and actor user; nevertheless, as many user profiles or sub-types as necessary may be added. Observational methods, interviews, focus groups, and role play among others can be used for this study [73]. Due to these, context characteristics, opportunities, and problems as well as user qualities, capacities, behaviour models, and desires can be extracted. To synthesise the information collected, some tools such as the ‘person-method’, user targets, or archetypes can be applied [74]. Moreover, in terms of use, developers can establish analogies with related products to approach the user’s base knowledge.

### 3.3. Step 3: Service Design

This step will be included if the product is considered a service, a growing area for MoCap; in this case, service design tools [75,76] would be an indispensable basis for the project. One of the most interesting methods is ‘Blueprint’ [77], a design tool and dialogue and training media for professional users, because it marks the necessary key moments in the service for each of the users involved through a temporary schematisation.

In a MoCap-wearable service, the key moments or actions that are usually carried out are the following: (1) explanation to the actor user; (2) device body placement; (3) anatomical calibration; (4) capture; (5) data and result generation; (6) data processing (by software and/or by user); and (7) final actions (e.g., medical reports, real-time feedback, etc.). This generic use protocol must be considered in the physical device design and if applicable, in the service definition. It will influence both users experience and, consequently, the final solution success.

### 3.4. Step 4: User Interaction

User interaction flows are defined in the Figure 6 diagram by arrows for all items that relate to users. These interaction lines can be unidirectional (feedback or biofeedback) or bidirectional (information exchange), which may vary depending on the device, the user, and the context.

In the early design iterations, these lines can be used to define some needs and specifications, based on the work already done; thus, a relational needs methodology can be followed. This tool is applied in cases like ours, where it is intended to design solutions that involve relationships between several users to detect those needs that each specific user has with respect to the others [78]. Here, it can be proposed to apply in the rest of the items, contemplating the human-machine and machine-machine relations.

With respect to human-machine interactions, as detailed in Section 2.2, the system interaction with the professional user can be bidirectional, from devices and from DPP, including interaction with devices regarding proper placement, maintenance, or connection and with DPP regarding capture configuration of possible incidents, annotation, or result study, analysis, and interpretation.

The human-machine interaction with the actor user are typically unidirectional, from the device and/or the DPP; it can be either as feedback or as biofeedback [63] and in a visual, auditory, or haptic way, showing different events like movement changes on a screen with a virtual scene, or the start and end sounds, among others. In design, it will be important to consider the elements that are necessary to allow and favour the mentioned user interaction.

### 3.5. Step 5: Technology

It is necessary to study the technological content, as it affects the physical product conception. To do this, it is proposed to approach it through some points, which, despite being presented sequentially, will require iterations due to their strong interrelation between them and with the rest of the sections:

- Make decisions about the technology type to be used (described in Section 2.3.1): optical, inertial, or even other MoCap technologies depending on the application. Thus, we can find solutions such as the one proposed by Shiratori et al. [64], which uses cameras fixed on the body.
- Define the main electronics that are needed, for which the electronic building blocks can be made and apply electronic design methodologies focused on designers or multidisciplinary teams, as Blanco et al. proposed [66]. Defining the building blocks (Figure 4) is necessary to ensure product viability, and to anticipate factors like the space that can occupy the electronics or the associated requirements and restrictions; for example, for IMU design, zones free of ferromagnetic materials and a minimum distance from the human body are required to not alter the magnetic fields and facilitate radiofrequency communications. The main blocks in the case of MoCap-wearables devices would be (Section 2.3.2) communication (dashed line of the schematic), storage, battery, intelligence, and interface (defined in Step 4). Note that, to be able to define some points of the electronics, it will be necessary to at least have selected the DPP that will process and register the data (Step 8) because the DPP will also influence the communications, functionalities, size of components, etc.
- Select the required number of MoCap-wearable devices to be placed on the body and the most appropriate interconnection between them (Section 2.3.3): full body suits, wired independent elements, or wireless devices.

### 3.6. Step 6: Body Attachment

It is necessary to consider the body attachment, a key factor in the design of wearables. At this point, the positioning and the attachment method must be determined. In relation to the positioning, the exact area must be determined, explaining the reasons for its selection, and investigating and testing the best rules to help the professional user determine its correct location. Regarding the attachment method, the proposed decision table can be used (Table 3), in which more attachment methods can be included as this scope progresses. In any case, prototyping at this stage will be crucial.

### 3.7. Step 7: Device Physical Properties

It is also required to define the physical MoCap-wearable device properties. For this purpose, criteria are presented in Section 2.5 related to shape, dimensions, weight, flexibility, material, comfort, psychological aspects, and aesthetics, which are crucial factors to achieve the necessary precision measurement for good user adaptation.

### 3.8. Step 8: DPP

Having selected the DPP product to perform processing, at this stage, it is not necessary to define the interaction elements because they are given by the product. Thus, this stage requires the software design, which, according to the purposes of each MoCap application, will utilise data processing and will communicate the appropriate and accurate information to carry out the user interaction. For this design, software development and interaction methodologies [62] can be followed. The development of this point depends on the system purpose, which, due to Octopus, is expected to extend to other areas not yet explored.

### 3.9. Case Study, Methodology Assessment

To illustrate the methodology and assess its applicability, a case study is included in Table 4, where the basic requirements of a shoulder rehabilitation service for elderly individuals are defined.

**Figure 7:**
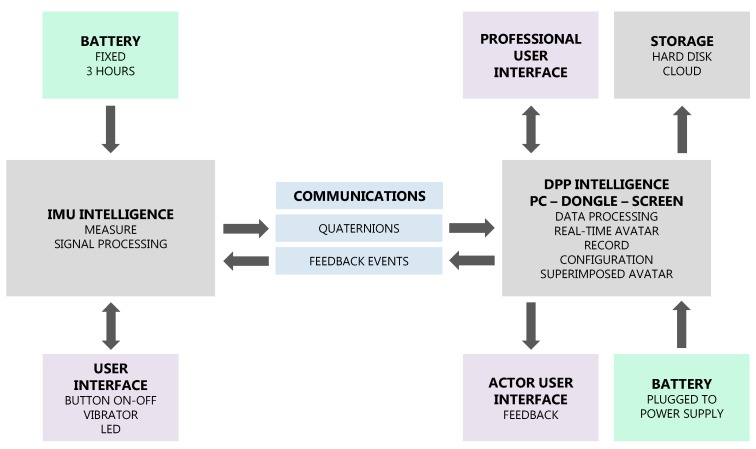
Case study building blocks.

The example summarised in Table 4 and shown in the building-block scheme (Figure 7) considers the main requirements for the case of a rehabilitation service. It has been observed that the structuring carried out systematises the development process from the design goal. In the absence of being applied in more cases, it is considered to be a useful tool for new or improved product development.

## 4. Conclusions

This article presents a problem about the MoCap-wearable systems that affects product and service designers and electronics developers. This problem is related to the difficulty of considering all human, ergonomic, technological, or material factors, among others. Thus, MoCap-wearable designs require studying the following critical aspects that have been identified: context, user interaction, technology, body attachment, and physical property factors.

In response to this problem, a bibliographical and representative commercial product review has been carried out, which allowed extracting the factors directly related to design requirements, which have been collected and ranked. As a result, the Octopus methodology is proposed, which aims to help the MoCap-wearable system requirement extraction process, due to the factor schematisation and a visual representation that allow this to be studied and evaluated sequentially.

Octopus begins by studying the context to later develop the product or service. The method allows the creation of systems aimed towards MoCap from one, several, or all parts of the body. It has several characteristics that imply an improvement in this type of product design process, as follows:

- It is flexible and adaptable. Without a closed and immovable scheme that limits creativity, the design team, which is necessarily multidisciplinary, can eliminate or add factors and elements according to each case.
- The tool makes the job easier. Due to the visual representation of Figure 6 and the different steps proposed, it is expected to improve organisation, structuring, synthesis, and facilitate decision making, providing a global view of all the necessary factors.
- Due to the case study, it has been observed that the tool allows us to generate innovative ideas in an effortless way and to consider the main specifications and design problems.
- It is a communication tool between professionals or researchers from different disciplines involved in the design team, illustrating the common objectives to be fulfilled, mapping the status of the project, and allowing the assessment and recognition of the contributions of each of the team members.

Due to the simplification of the MoCap-wearable system creation process, this article introduces different fields and possibilities:

- In the current context of the technological progress, electronics miniaturisation, cost reduction, and the IoT, Octopus can contribute to facilitating the success of new products aimed at the MoCap area. It is expected that developed MoCap-wearable systems will be more suited to users and environments, so that MoCap can be used more extensively solving current problems in existing applications and allowing its implementation in more and new ones.
- In relation to this search for new applications, it is observed that the assumption of the MoCap as a service can be an improvement and an opportunity to cover real needs.
- It is expected that the study increases knowledge of optical rigid body MoCap systems that, according to Baker et al. [22], have been largely ignored in the literature as of 2006, and it has been verified in further review that it has not increased in the last years.
- MoCap-wearables devices can be considered products of high or very high complexity in relation to the design requirements, so the study can also be extrapolated to other wearables and to other areas with less complexity. In fact, the described factors can be used not only for advanced technological devices but also for other, more basic products that need to be precisely and comfortably placed on the body.

As future work, the methodology should be implemented in more cases to improve it. Additionally, it is proposed to create objective methods to adequately evaluate the prototypes developed by operating in real situations, mainly in relation to the union attachment. In this way, it is proposed to investigate new types of body attachment. This may be interesting to study regarding the behaviour of the skin with the body movement and the design of the contact surfaces, considering anthropometric and morphological factors of the subject under study. In addition, there is no doubt that there is a latent need to look for new MoCap applications and areas, improving the movement and consequently the quality of life of more sectors of the population.

## Figures and Tables

**Table 1:** Product and service market examples.

Full Body Systems MoCap				Wearable Products		
Wireless Inertial Products	Wired Inertial Products ^1^	Optical Products	Services	Head	Chest	Extremities
Noraxon [32]	Perception Neuron [33]	Natural point [14]	MySwing [34]	Alex Posture [35]	Araig [36]	LEO Fitness Intelligence [37]
Notch [38]	Rokoko studios [39]	Vicon [40]	Run3D [41]	Google Glass [42]	MC10 [43]	Quell relief [44]
Perception Legacy [45]	Shadow [46]		Imaginarium Studios [47]	Jolt Sensor [48]	SenseOn [49]	Sensoria fitness [50]
Stt-Systems [51]	Technaid [52]			Melon Headband [53]	Tesla Suit [54]	Thalmic Labs [55]
Trivisio [15]	Xsens (suit) [56]			Reebok checklight [57]	UpRight [58]	
Xsens [56]				Thync [59]		

^1^ Wired sensor to sensor products, but wireless communication with data processing point (DPP).

**Table 2:** Link between some MoCap environments and their users.

	Environment	Professional User	Actor User
**Medicine**	Diagnosis	Doctor	Patient
	Rehabilitation	Doctor, Physiotherapist	Patient
	Forensic	Forensic Doctor	Injured (may be uncooperative)
**Sports**	Performance	Coach, Physiotherapist	Athlete
	Rehabilitation	Coach, Physiotherapist	Athlete
**Animation Simulation**	Professional simulation	Coach, Technician, Others	Athlete, Military, Others
	Video game	Player	Player
	Cinema/theatre	Director, Technician, Others	Performer
**Research**	Laboratory	Developer, Researcher	Unknown

**Table 3:** MoCap-wearable device body attachment methods valued by factors (1—worst, 3—best).

Attachment Method	Area Selection	Preparation Speed	Washing	Adapt-Ability	Fungible Restrictions	Union Distribution	Union Strength	Result (R)
Weight (W)	(5) ^1^	(4) ^1^	(9) ^1^	(10) ^1^	(2) ^1^	(8) ^1^	(8) ^1^	-
Closed elastic tape ^2^	3	1	1	2	3	3	3	(102) ^1^
Open elastic tape ^2^	3	2	1	3	3	3	3	(116) ^1^
Garment (fixed pockets, clips) ^2^	1	2	1	1	3	3	3	(86) ^1^
Garment (Velcro areas) ^2^	3	2	1	1	3	3	3	(96) ^1^
Commercial electrode ^3^	3	3	3	3	1	1	2	(110) ^1^
Custom electrode ^3^	3	3	3	3	1	2	2	(118) ^1^
Double-sided tape ^3^	3	2	3	3	1	1	2	(106) ^1^
Bandage kinesiotape ^3^	3	1	3	3	1	3	3	(126) ^1^
Semi-rigid (bracelet, flaps) ^4^	2	3	3	2	3	3	1	(107) ^1^

^1^ Example of scoring for a sanitary elderly rehabilitation application; ^2^ Fabric fixing supports; ^3^ Disposable adhesive fixing supports; ^4^ Semi-rigid fixing supports.

**Table 4:** Case study, shoulder rehabilitation service for elderly individuals.

**Step 1: Design Goal**
Shoulder rehabilitation service for elderly in private clinics.
**Step 2: Context Study**
**Professional User Target:**
Occupation: Physiotherapist. Age: 35. Technology level: Medium—High.
Verbatim: ‘I am a person who wants to improve and learn every day in my work. I have little time since I attend about six patients a day in 45-min sessions’.
**Actor User Target (patient):**
Occupation: Retired. Age: 78. Technology level: Low.
Verbatim: ‘At my age, I appreciate tranquillity and patience; it makes me feel safer’.
**Environmental Characteristics:**
Indoor, bright, hygienic, and clean. Furniture: work tables, chairs, and stretchers.
**Step 3: Service Design**
Explain the test to the patient. Place the devices on the patient and warm up. Record the target movement cyclically (e.g., flexor-extension or internal-external rotation), while the physiotherapist mobilises the patient’s shoulder according to the appropriate rehabilitation schedule. The patient repeats the movement alone while receiving biofeedback from the recorded motion. Report of the results (success or failure regarding the recorded movement cycle).
**Step 4: User Interaction**
**DPP—Professional Interaction:**
Allow to start and pause motion recording (possibility to do it remotely).
Set the number of repetitions of the exercise and grade the threshold to consider whether the movement is correct for rehabilitation purposes in the session.
Observe the results.
**DPP—Patient Interaction:** Display an avatar that moves in real time according to the patient’s movement. Display a superimposed avatar that plays the pre-recorded motions during the professional’s mobilisation. Use gamification techniques to facilitate tracking of target movement.
**Device—Professional Interaction:** During the recording period, the user interacts with the devices through the visual sense: green LED—transmitting and no movement recorded, red LED—recording, and blue LED—there is movement recorded.
**Device—Patient Interaction:** During the task of repetitive movements, the patient interacts with the haptic sense (vibration) to indicate breaks, repetitive failures, phase changes, etc.
**Step 5: Technology**
**Technology Selection:** IMU devices are used. No special lighting or space requirements are necessary. During the physiotherapist manipulation, there are no problems with marker occultations.
**Building Blocks:** Shown in Figure 7.
**Number of Sensors:** Five are placed if a single upper extremity is examined and seven for both.
**Connection:** The sensors are wireless and connect to the DPP via Wi-Fi.
**Step 6: Body Attachment**
**Placement:** Sacrum, on vertebra D2, the upper area of the head, outside of the arm just above the elbow, and on the forearm on the upper face of the wrist.
**Attachment Method:** Device on the skin, attached to the body by pre-cut kinesio-tape bands with hole to protrude the device and leave the LED visible. (In case of excessive hair, this would be removed. The area is cleaned with disinfectant). The choice of this attachment method is justified in the Table 3 example.
**Step 7: Device Physical Properties**
**Shape:** Oval, rounded edges with wings in the skin contact area, where the tape is fixed.
**Dimensions:** 4 × 3 cm.
**Weight:** 40 grams per sensor.
**Housing material:** Rigid plastic and resistant to humidity. It is painted with silicone texture for easy sanitising.
**Colour:** It is white, adapts to the environment, and transmits appropriate values.
**Step 8: DPP**
**Elements:** Computer, Wi-Fi dongle for communications, and an external monitor.
**Basic software operation:** It has two modes, the configuration for the professional user and the display for the patient user. It calculates the hits and misses regarding the target movement and displays them in real time. It connects to the Internet to store data in the cloud at the end of the session.

## Abbreviations

MoCap	Motion Capture
IoT	Internet of Things
IMU	Inertial Measurement Unit
DPP	Data Processing Point
LED	Light-Emitting Diode
GUI	Graphic User Interface
RMS	Root Mean Square
SD	Secure Digital
LAN	Local Area Network
PAN	Personal Area Network
